# Adjuvant vitamin C in cardiac arrest patients undergoing renal replacement therapy: an appeal for a higher high-dose

**DOI:** 10.1186/s13054-018-2115-9

**Published:** 2018-08-16

**Authors:** Patrick M. Honore, David De Bels, Thierry Preseau, Sebastien Redant, Rachid Attou, Herbert D. Spapen

**Affiliations:** 1 ICU Department Centre Hospitalier Universitaire Brugmann, Place Van Gehuchtenplein,4, 1020 Brussels, Belgium; 2 Emergency Department Centre Hospitalier Universitaire Brugmann, Brussels, Belgium; 3 Universitair Ziekenhuis Brussel, VUB University, Brussels, Belgium

We read with interest the excellent review of Spoelstra-de Man et al. which focused on the potential benefit of adjuvant vitamin C (vit C) therapy in ischemia-reperfusion injury [1]. Following an exhaustive in-depth analysis of the impressive experimental, clinical, and safety record of vit C, the authors plead for a randomized controlled clinical trial assessing the effect of early, high-dose (i.e., at least 3 g/day), intravenous vit C administration in post-cardiac arrest patients.

About half of the patients may develop acute kidney injury stage ≥ 1 within 2 days after cardiac arrest and 20 to 60% will require renal replacement therapy (RRT) [2]. Vit C has a molecular weight of 176 Dalton and is thus exposed to significant clearance during RRT. Intermittent hemodialysis as well as continuous RRT (CRRT) are indeed associated with a 50% reduction of plasma ascorbate and vit C levels [3–5]. Diffusion and convection account for two-thirds and one-third, respectively, of the vit C loss [3]. A 3 g daily vit C dose, therefore, is by no means guaranteed to cover the acute need in post-cardiac arrest patients initiated on (C)RRT. Vasopressor-dependent subjects in particular may benefit from increased dosing because vit C has been shown to support endogenous vasoactive catecholamine synthesis. Awaiting solid pharmacological data, we propose to supplement post-cardiac arrest patients not treated with CRRT with 6 g vit C daily. If CRRT is running, the dose should be increased to 12 g. We fully agree with Spoelstra-de Man et al. to administer vit C as early as possible (i.e., before intensive care admission) and to continue treatment for a short period of time.

## Abbreviations

CRRT: Continuous renal replacement therapy
RRT: Renal replacement therapy
Vit C: Vitamin C
